# Investigating strategies to enhance access to HIV services among children in Malawi: A multifaceted research approach, study protocol

**DOI:** 10.1371/journal.pone.0357408

**Published:** 2026-09-03

**Authors:** Bilaal Wilson Matola, Juliet Charity Yauka Nyasulu, Edward Nicol

**Affiliations:** 1 Division of Health Systems and Public Health, Department of Global Health, Faculty of Medicine and Health Sciences, Stellenbosch University, Stellenbosch, South Africa; 2 Department of HIV, STIs and Viral Hepatitis, Ministry of Health, Lilongwe, Malawi; 3 AFRIQUIP, Health Systems Strengthening, Johannesburg, South Africa; 4 Burden of Disease Research Unit, South Africa Medical Research Council, Cape Town, South Africa; University of Ottawa, CANADA

## Abstract

**Background:**

Despite major progress in HIV epidemic control, children in sub-Saharan Africa, particularly in Malawi, continue to experience poorer HIV outcomes than adults. While Malawi has made substantial progress toward the UNAIDS 95-95-95 targets, only 55% of children living with HIV are virally suppressed compared with 87% of adults. This study aims to identify and develop strategies to improve children`s access to HIV services in Malawi.

**Methods:**

Guided by the Socio-Ecological Model and the HIV care cascade framework, this multi-method study comprises four sub-studies: (1) secondary analysis of national HIV program data and spectrum estimates (2012–2023) to assess trends and progress toward the 95-95-95 targets; (2) a scoping review of health system interventions that improve paediatric HIV service access in sub-Saharan Africa (2015–2025). (3) a modified e-Delphi process with 15–30 experts to develop a context-appropriate intervention package and (4) a feasibility and acceptability assessment of the package at Kabudula Community Hospital, Lilongwe, Malawi, involving healthcare workers, community health workers, and caregivers using questionnaires and qualitative interviews.

**Ethical considerations:**

Ethical approval will be obtained from Stellenbosch University Health Research Ethics Committee (HREC) and the Malawi National Health Sciences Research Ethics Committee. Written informed consent and confidentiality will be ensured.

**Conclusion:**

This study will identify gaps in HIV care access among children, synthesize evidence on effective interventions, and develop a feasible, context-specific intervention package to strengthern children`s access to HIV services and accelerate Malawi`s progress toward achieving UNAIDS 95-95-95 targets.

## Introduction

The HIV/AIDS epidemic remains a major global public health challenge despite remarkable progress in prevention, and treatment over the past three decades. Sub-Saharan Africa continues to bear the greatest burden of the epidemic, accounting for nearly two-thirds of all people living with HIV (PLHIV) worldwide [1,2]. Malawi, a low-income country in this region, remains one of the countries most affected by HIV. In 2024, the country reported an HIV prevalence of approximately 7.3% among adults aged 15 years and older and 0.6% among children under 15 years [3]. Although the scale up of HIV interventions has resulted in substantial declines in new infections and AIDS related mortality since the mid-1990s [1], important disparities in HIV outcomes persist across population groups.

Global progress has been driven by coordinated efforts to expand access to HIV prevention, treatment and care services. Since its establishment in 1996, the Joint United Nations Programme on HIV/AIDS (UNAIDS) has led the global response to HIV with the goal of ending AIDS as a public health threat by 2030 [4]. The UNAIDS Fast-Track strategy, launched in 2014, introduced the 95-95-95 targets, which aim for 95% of all people living with HIV to know their HIV positive status, 95% of those diagnosed with HIV to receive sustained antiretroviral therapy (ART), and 95% of those on treatment to achieve viral load suppression by 2025 [4].

Globally, important gains have been made toward these targets. By 2023, 86% of people living with HIV globally knew their HIV status, 77% were receiving ART, and 72% had achieved viral suppression [1]. In sub-Saharan Africa, the corresponding figures were 93%, 83% and 78%, respectively [1].

Malawi has made considerable progress towards these targets through its public health approach to HIV service delivery. Since adopting this approach in 2004 and implementing the UNAIDS 90-90-90 targets in 2015, later transitioning to the 95-95-95 targets in 2020, the country has substantially expanded HIV testing and treatment services [5]. By the end of the 90-90-90 implementation period, 88.4% of people living with HIV were aware of their HIV positive status, 97.8% of those diagnosed were receiving treatment, and 96.9% of those on treatment had achieved viral suppression [6]. These achievements demonstrate the strength of Malawi`s national HIV programme.

Despite this progress, children continue to experience poorer outcomes than adults across the HIV care continuum [7,8]. In this study, children are defined as individuals younger than 15 years of age, consistent with the definitions adopted by the World Health Organization [9] and the Malawi National HIV Programme. The terms children and paediatric are used interchangeably throughout the manuscript. Children experience lower rates of HIV diagnosis, delayed ART initiation, poorer retention in care, suboptimal treatment adherence, and lower viral suppression than adults [10–15]. Access to HIV services is defined as the ability of children to obtain timely, equitable, and continuous HIV services across the care continuum, including HIV testing, diagnosis, linkage to care, ART initiation, retention in care, viral load monitoring, and achievement of sustained viral suppression [16]. Improving access therefore extends beyond the physical availability of services to include their accessibility, acceptability, affordability, and continuity throughout treatment. Achieving and sustaining epidemic control among children is critical, not only for improving child health outcomes but also for preventing HIV transmission and reducing the risk of drug resistance [17].

These disparities are particularly evident in Malawi. While viral suppression among adults reached approximately 87% by the end of 2023, suppression among children was only about 55% [18]. Children continue to experience losses at every stage of the HIV care continuum, from HIV diagnosis and linkage to care to treatment initiation, retention, and long-term viral suppression [19]. These persistent gaps threaten Malawi`s progress towards achieving the UNAIDS 95-95-95 targets and underscore the need for strategies specifically tailored to children`s needs.

Numerous health system interventions have been implemented globally and in Malawi to strengthen HIV service delivery, including decentralisation of HIV services [20], task shifting and task sharing [20], universal “test and treat” policies [21], integration of HIV services into primary healthcare [22,23], differentiated service delivery models [24], community-based HIV programmes [25], strengthened monitoring and evaluation systems [20], as well as innovative financing through global partnerships [26].

Although these interventions have substantially improved HIV outcomes at the population level, children continue to lag because they face unique barriers, including delayed early infant diagnosis [27], poor linkage to treatment [28], inconsistent retention in care, dependence on caregivers, stigma, long travel distances [29], financial constraints, and socialeconomic disadvantage.

Evidence suggests that interventions such as task shifting to lay health workers [30–33], integration of paediatric HIV services with maternal and child health services [25,34,35], expansion of point-of-care diagnostic technologies [36–42], decentralisation of ART services and differentiated service delivery models, have demonstrated potential for improving treatment uptake and retention among children [43–49].

However, the evidence remains fragmented and highly context-specific, with limited sysnthesis of which interventions are most effective for improving access to HIV services among children in sub-Saharan Africa. Furthermore, few national studies have comprehensively analysed routine programme data to identify where children are being lost along the HIV care continuum in Malawi. Consequently, policymakers have limited evidence to guide the development of comprehensive and contextually appropriate strategies to improve HIV service delivery in children.

Addressing these gaps requires more than a single methodological approach. National HIV epidemiological analyses can identify where service delivery gaps occur but cannot determine which interventions are most effective. Conversely, evidence from published studies can identify promising interventions but may not adequately reflect the Malawian context. Moreover, evidence-based interventions require prioritisation by stakeholders and assessment of their feasibility before implementation. An integrated research approach is therefore needed to combine epidemiological evidence, evidence synthesis, stakeholder consensus, and implementation assessment.

**Fig 1:**
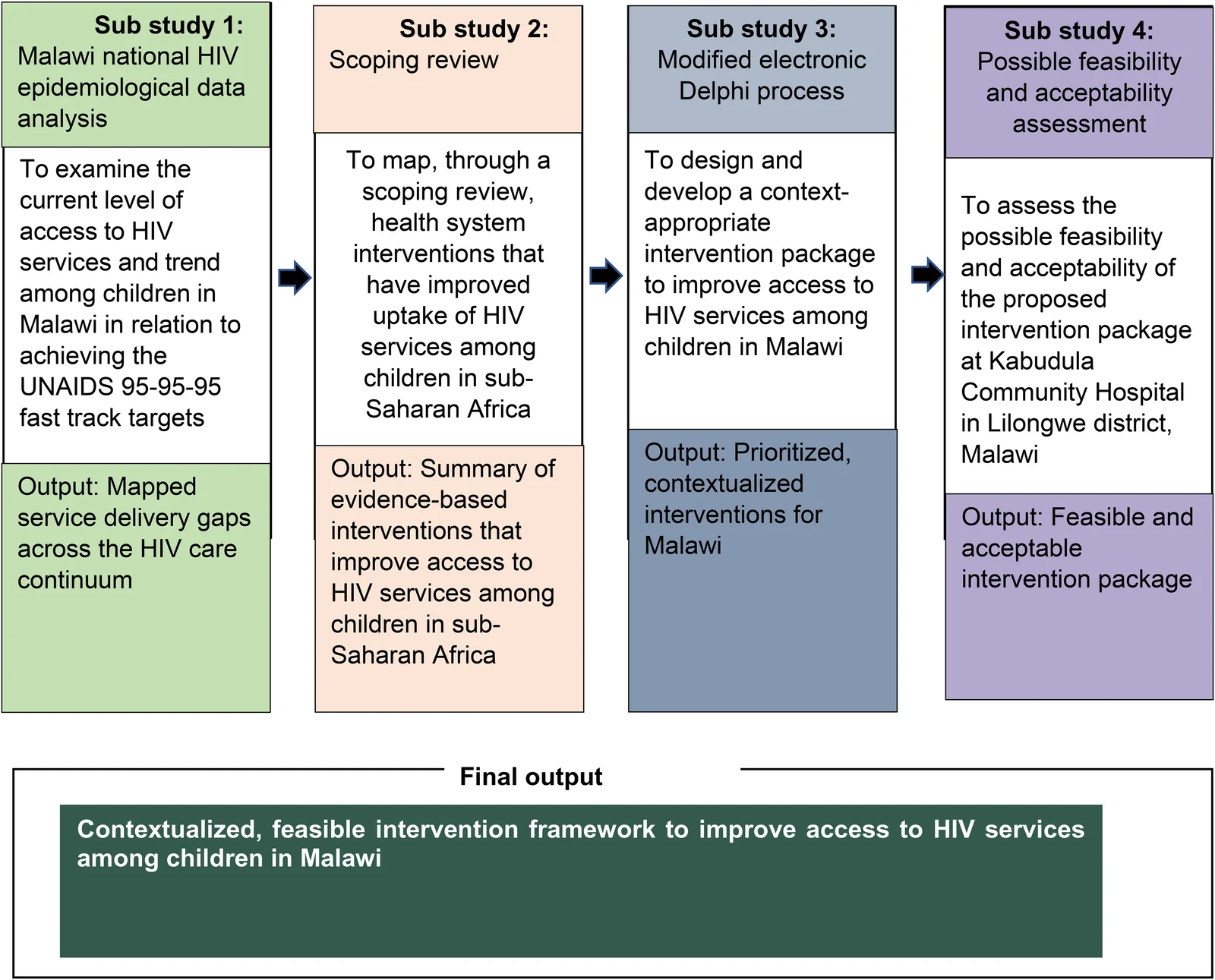
Conceptual workflow of the study. Illustrates the conceptual workflow of the study and the relationship between the individual sub studies.

Accordingly, this study adopts a sequential multi-method design comprising four complementary methodological components. First, a national epidemiological analysis will identify service delivery gaps across the paediatric HIV care continuum. Second, a scoping review will synthesise evidence on interventions that have improved access to HIV services among children in sub-Saharn Africa. Third, a modified Delphi process will engage key stakeholders to contextualise and prioritise these interventions for Malawi through expert consensus. Finally, a feasibility assessment will evaluate the acceptability, practicality, and implementation potential of the resulting intervention framework. Fig 1 summarises the interplay between the four sub studies.

Together, these four components provide a coherent pathway from identifying service delivery gaps, to identifying evidence-based solutions, adapting them to the local context, and evaluating their readiness for implementation. By integrating epidemiological analysis, evidence synthesis, stakeholder consensus, and feasibility assessment, this study aims to develop an evidence-informed and contextually appropriate framework for improving access to HIV care services among children in Malawi. The findings will inform policy and programme implementation, strengthen children HIV service delivery, and ontribute to achieving equitable progress towards the UNAIDS 95-95-95 targets and ending AIDS as a public health threat by 2030. The main research question guiding this study is: How can an evidence-informed and contextually appropriate framework of strategies be developed to improve access to HIV care services among children in Malawi? The broad objective of this study is to develop and assess the possible feasibility of an evidence-informed, contextually appropriate framework for improving access to HIV services among children in Malawi. The following are the specific objectives: 1. To assess levels and trends in access to HIV services among children in Malawi in relation to progress toward the UNAIDS 95-95-95 targets. 2. To map, through a scoping review, health system interventions that have improved uptake of HIV services among children in sub-Saharan Africa. 3. To design and develop a context-appropriate intervention package to improve access to HIV services among children in Malawi. 4. To assess the possible feasibility and acceptability of the proposed intervention package at Kabudula Community Hospital in Lilongwe district, Malawi.

## Materials and methods

This study will use a sequential multi-method study design [50]. The study comprises four interrelated sub-studies in line with the objectives. Below are the details:

### Sub-study 1: To examine the current level of access to HIV services and trend among children in Malawi in relation to achieving the UNAIDS 95-95-95 fast track targets

#### Study design.

This study employed a descriptive quantitative design [51] using secondary data derived from routinely collected Malawi national HIV programme database and UNAIDS spectrum estimates. The study systematically analysed existing programme data to describe temporal trends, quantify progress, and identify gaps in HIV service delivery across the HIV care continuum. Statistical analyses were conducted to objectively assess Malawi`s progress towards achieving the UNAIDS 95-95-95 targets, with particular emphasis on examining disparities in HIV testing, antiretroviral therapy (ART) coverage, and viral load suppression across key population subgroups, including age and sex. The use of routinely collected national data enabled a comprehensive evaluation of programme performance under real-world implementation conditions and provided evidence to inform policy and programme improvement.

#### Data sources.

This study utilized secondary data obtained from Malawi’s national HIV programme and UNAIDS spectrum estimates. Routine programme data were extracted from the Department of HIV/AIDS Management Information System (DHAMIS), the national repository for HIV programme data maintained by the Ministry of health. DHAMIS receives routinely reported, aggregated data from all health facilities providing HIV prevention, testing, treatment, and care services across the country. The analysis included programme data collected between 2012 and 2023, enabling the assessment of longitudinal trends in HIV service delivery and programme performance. To complement the routine programme data, UNAIDS spectrum estimates were used to obtain epidemiological parameters that are not routinely captured through health facility reporting, including national estimates of the total number of people living with HIV (PLHIV). Spectrum is a globally recognized mathematical modelling tool that generates country-specific HIV estimates by integrating surveillance, demographic, and programme data [52]. The combined use of routine programme data and spectrum estimates provided a more comprehensive assessment of Malawi`s progress towards the UNAIDS 95-95-95 targets by integrating observed programme performance with modelled population-level estimates.

#### Study variables.

The primary outcome variables were the three indicators of the UNAIDS 95-95-95 HIV care cascade: HIV status awareness, antiretroviral therapy (ART) uptake, and HIV viral load suppression. These indicators were assessed longitudinally to evaluate trends in HIV programme performance between 2012 and 2023. HIV status awareness was defined as the proportion of people living with HIV (PLHIV) who were aware of their HIV-positive status. ART uptake was defined as the proportion of individuals diagnosed with HIV who were receiving antiretroviral therapy. Viral load suppression was defined as the proportion of individuals receiving ART who achieved viral suppression, as defined by the Malawi National HIV Programme and UNAIDS monitoring guidelines. To assess equity in HIV service delivery, all outcome variables were disaggregated by age and sex. Age was categorized into children (under 15 years) and adults (15 years and older), consistent with national HIV programme reporting, while sex was categorized as male and female. Stratified analyses were undertaken to identify temporal differences in programme performance across demographic subgroups and to quantify disparities in access to HIV diagnosis, treatment and treatment outcomes. This disaggregation enabled the identification of population groups experiencing suboptimal performance across the HIV care continuum, thereby informing targeted policy and program interventions.

#### Data analysis tools.

Data were pre-processed through systematic cleaning, validation, and consistency checks to ensure completeness, accuracy and internal consistency prior to analysis. initial data management, including data verification, coding, and tabulation, was conducted using Microsoft Excel. Statistical analyses were performed using Stata version 15.1.

#### Statistical analysis.

Descriptive statistical analyses were conducted to summarize the characteristics of the study population and describe temporal trends in the three UNAIDS 95-95-95 indicators: HIV status awareness, antiretroviral therapy (ART) coverage, and viral load suppression. Categorical variables were summarized using frequencies and proportions, while trends in programme performance from 2012 to 2023 were presented graphically and in tabular form. Comparative analyses were undertaken to assess differences in HIV programme outcomes across demographic subgroups. For the 2023 cross-sectional analysis, proportions of HIV status awareness, ART coverage, and viral load suppression were compared by age group (children <15 years and adults ≥15 years) and sex. Pearson`s chi-square test was used to compare proportions between groups, while Fisher`s exact test was applied where expected cell counts were insufficient to satisfy the assumptions of the chi-square test. Corresponding 95% confidence intervals were reported for key proportions and estimated differences where appropriate. All statistical tests were two-sided, and statistical significance was determined using an alpha level of 0.05 (p < 0.05).

#### Timelines.

The UNAIDS 95-95-95 targets were originally set for achievement by December 2025. To maintain relevance to this timeline, this sub-study was completed and its findings published in May 2025. The full publication and data set are publicly accessible online at the following link: https://www.tandfonline.com/doi/abs/10.2989/16085906.2025.2477090.

### Sub study 2: To describe the landscape of health system interventions that have improved access to HIV services among children in sub-Saharan Africa

#### Study design.

This study will employ a scoping review design [53] to systematically map and synthesise the existing evidence on health system interventions that improve access to HIV services among children in sub-Saharan Africa. The review will be conducted in accordance with the Joanna Briggs Institute (JBI) methodology for scoping reviews, which represents the current international standard for the conduct of scoping reviews and builds upon the foundational framework proposed by Arksey and O’Malley and its subsequent refinements [54,55]. Consistent with the JBI methodology, the review will follow a structured and iterative process comprising: (i) defining the review objectives and research question; (ii) developing and implementing a comprehensive search strategy; (iii) identifying and selecting eligible studies based on predefined inclusion and exclusion criteria; (iv) extracting and charting data using a standardised data extraction form; and (v) collating, summarising, and presenting the findings in relation to the review objectives. Throughout the review process, methodological decisions will be documented to ensure transparency, reproducibility, and methodological rigour. The review findings will be reported in accordance with the Preferred Reporting Items for Systematic Reviews and Meta-Analysis Protocols Extension for Scoping Reviews (PRISMA-ScR), which provides guidance for the transparent and comprehensive reporting of scoping review methods and findings [56]. Adherence to the JBI methodology and PRISMA-ScR reporting standards will enhance the methodological quality, transparency, and reproducibility of the review.

#### Patient and public involvement.

This study will be conducted using previously published data and will not involve direct participation from patients at any stage. No patients will be recruited, interviewed, or subjected to any interventions as part of this research. Additionally, patient input will not be sought in the design, data collection, analysis, or dissemination of the study findings.

**Stage 1: Formulating the research question:** An iterative approach, consistent with contemporary scoping review methodology was used to progressively refine the research question through ongoing engagement with co-investigators. This iterative refinement ensured conceptual clarity, scope alignment, and methodological coherence throughout the review process. The overarching review question is: Which health system interventions have been implemented to improve access to HIV services among children in sub- Saharan Africa?

The review is further structured by the following specific questions:

1. What types of health system interventions have been implemented to improve access to HIV services among children in sub-Saharan Africa?
2. What are the key characteristics of these interventions, including delivery platforms, target populations, and implementation strategies?
3. What outcomes have been reported in relation to child access to HIV services following implementation of these interventions?

**Stage 2: Identifying relevant studies:** A comprehensive literature search will be conducted to identify studies reporting health system interventions that improve access to HIV services among children living with HIV in sub-Saharan Africa. The search strategy will be developed in consultation with an experienced health sciences librarian to maximise its sensitivity and reproducibility. Electronic databases to be searched will include PubMed/MEDLINE, Embase, WHO Global Index Medicus, Google Scholar, and Web of Science. In addition, backward and forward citation tracking of all included studies and relevant review articles will be undertaken to identify additional eligible studies not retrieved through database searching.

The review focuses on interventions implemented following the introduction of the UNAIDS Fast-Track (90-90-90) targets in 2014, which marked a shift towards targeted strategies for improving performance across the HIV care cascade [4]. Accordingly, studies published between 1 January 2015 and 31 July 2025 will be considered for inclusion.

The search strategy will be developed using the Population-Concept-Context (PCC) framework, which is recommended by the Joanna Briggs Institute for scoping reviews. Search terms will be identified through an iterative process involving the review question, Medical Subject Headings (MeSH), database specific subject headings, and relevant free text keywords. Search terms will encompass three domains: (i) Population (e.g., “child*”, “paediatric HIV”, “pediatric HIV”, “infant*”, “HIV-exposed children”); (ii) Concept (e.g., “health system*”, “health service*”, “intervention*”, “service delivery”, “access”, “linkage”, “retention”, “viral suppression”); and (iii) Context (e.g., “sub-Saharan Africa” and the names of individual countries within the region). Although terms such as “adolescent” may be included during the initial search to increase search sensitivity and account for studies that report combined paediatric populations, only data relating to children aged 0–14 years will be eligible for inclusion during study selection.

Pilot searches will be conducted to refine the search syntax for each database, and the final search strategies will be adapted to the indexing requirements of individual databases. The complete electronic search strategy for each database will be provided to ensure transparency and reproducibility. Table 1 shows detailed search strategy for PUBMED.

**Table 1:** Detailed PubMed search strategy.

Component	Description
Review question	What health system interventions have been implemented to improve access to HIV services among children in sub-Saharan Africa?
Framework	Population–Concept–Context (PCC) framework as recommended by the Joanna Briggs Institute (JBI) for scoping reviews.
Population	Children (<15 years), including infants, children, adolescents, paediatric populations, and newborns living with or at risk of HIV infection.
Concept	Health system interventions implemented to improve access to HIV services, including interventions related to service delivery, health systems strengthening, health policy, healthcare accessibility, programme evaluation, and healthcare interventions.
Context	Countries in sub-Saharan Africa.
Database	PubMed
Publication period	1 January 2015 to 31 July 2025
Language	English
Search fields	Medical Subject Headings (MeSH) and Title/Abstract (tiab) fields.
Search approach	Controlled vocabulary (MeSH) and free-text terms were combined using Boolean operators. Synonymous terms within each concept were combined using OR, while the four concepts (HIV, health system interventions, children, and sub-Saharan Africa) were combined using AND. Truncation (*) was used where appropriate to capture variations of search terms.
Concept 1: HIV services	MeSH terms: “HIV Infections”[Mesh]Keywords: HIV, “HIV services”, “HIV care”, “HIV treatment”, “HIV testing”, “HIV diagnosis”, “HIV cascade”, “antiretroviral therapy”, ART
Concept 2: Health system interventions	MeSH terms: “Health Services”[Mesh], “Health Systems”[Mesh], “Health Policy”[Mesh], “Delivery of Health Care”[Mesh], “Health Services Accessibility”[Mesh], “Program Evaluation”[Mesh]Keywords: “Healthcare Interventions”, “health system strengthening”, “service delivery”, “healthcare access”, “health service intervention*”, “system intervention*”
Concept 3: Children	MeSH terms: “Child”[Mesh], “Infant”[Mesh], “Adolescent”[Mesh]Keywords: child*, pediatric*, paediatric*, adolescent*, infant*, newborn*
Concept 4: Geographic setting	MeSH term: “Africa South of the Sahara”[Mesh]Keywords: “sub-Saharan Africa”, Angola, Benin, Botswana, Burkina Faso, Burundi, Cameroon, Cape Verde, Central African Republic, Chad, Comoros, Congo, Côte d'Ivoire, Djibouti, Equatorial Guinea, Eritrea, Eswatini, Ethiopia, Gabon, Gambia, Ghana, Guinea, Guinea-Bissau, Kenya, Lesotho, Liberia, Madagascar, Malawi, Mali, Mauritania, Mozambique, Namibia, Niger, Nigeria, Rwanda, Sao Tome and Principe, Senegal, Seychelles, Sierra Leone, Somalia, South Africa, South Sudan, Sudan, Tanzania, Togo, Uganda, Zambia, and Zimbabwe.
Complete PubMed search string	(("HIV Infections"[Mesh] OR HIV[tiab] OR "HIV services"[tiab] OR "HIV care"[tiab] OR "HIV treatment"[tiab] OR "HIV testing"[tiab] OR "HIV diagnosis"[tiab] OR "HIV cascade"[tiab] OR "antiretroviral therapy"[tiab] OR ART[tiab]) AND ("Health Services"[Mesh] OR "Health Systems"[Mesh] OR "Health Policy"[Mesh] OR "Delivery of Health Care"[Mesh] OR "Health Services Accessibility"[Mesh] OR "Program Evaluation"[Mesh] OR "Healthcare Interventions"[tiab] OR "health system strengthening"[tiab] OR "service delivery"[tiab] OR "healthcare access"[tiab] OR "health service intervention*"[tiab] OR "system intervention*"[tiab]) AND ("Child"[Mesh] OR "Infant"[Mesh] OR "Adolescent"[Mesh] OR child*[tiab] OR pediatric*[tiab] OR paediatric*[tiab] OR adolescent*[tiab] OR infant*[tiab] OR newborn*[tiab]) AND ("Africa South of the Sahara"[Mesh] OR "sub-Saharan Africa"[tiab] OR Angola[tiab] OR Benin[tiab] OR Botswana[tiab] OR "Burkina Faso"[tiab] OR Burundi[tiab] OR Cameroon[tiab] OR "Cape Verde"[tiab] OR "Central African Republic"[tiab] OR Chad[tiab] OR Comoros[tiab] OR Congo[tiab] OR "Côte d'Ivoire"[tiab] OR Djibouti[tiab] OR "Equatorial Guinea"[tiab] OR Eritrea[tiab] OR Eswatini[tiab] OR Ethiopia[tiab] OR Gabon[tiab] OR Gambia[tiab] OR Ghana[tiab] OR Guinea[tiab] OR "Guinea-Bissau"[tiab] OR Kenya[tiab] OR Lesotho[tiab] OR Liberia[tiab] OR Madagascar[tiab] OR Malawi[tiab] OR Mali[tiab] OR Mauritania[tiab] OR Mozambique[tiab] OR Namibia[tiab] OR Niger[tiab] OR Nigeria[tiab] OR Rwanda[tiab] OR "Sao Tome and Principe"[tiab] OR Senegal[tiab] OR Seychelles[tiab] OR "Sierra Leone"[tiab] OR Somalia[tiab] OR "South Africa"[tiab] OR "South Sudan"[tiab] OR Sudan[tiab] OR Tanzania[tiab] OR Togo[tiab] OR Uganda[tiab] OR Zambia[tiab] OR Zimbabwe[tiab]))

Table 1 presents the detailed search strategy to be used to identify relevant studies in PubMed, including the search terms, Boolean operators, and search combinations applied.

The review will be restricted to studies published in English because of resource limitations for translation. This restriction may introduce language bias and result in the omission of relevant evidence published in other langauges; therefore, this limitation will be acknowledged when interpreting the findings.

***Eligibility criteria:*** Eligibility criteria will be established using the Population, Concept, and Context (PCC) framework: Population: Children (aged 0–14 years) living with HIV. Concept: Health system interventions designed to improve access to HIV services across the HIV care continuum including HIV testing, linkage to care, treatment initiation, retention in care, adherence, and viral load monitoring or suppression. Context: Healthcare facilities and community based service delivery settings in countries within sub–Saharan Africa.

The review will include peer-reviewed primary research studies employing quantitative, qualitative, or mixed-methods designs, including randomized controlled trials, quasi-experimental studies, cohort studies, case-control studies, cross-sectional studies, implementation studies, and qualitative research. Editorials, commentaries, opinion papers, conference abstracts without full-text publications, and narrative reviews will be excluded. Table 2 summarises the study eligibility criteria.

**Table 2:** Study eligibility criteria.

Eligibility domain	Criteria
Language	Studies published in English only
Population	Children aged 0–14 years living with HIV.
Concept	Health system interventions designed to improve access to HIV services across the HIV care continuum, including HIV testing, linkage to care, treatment initiation, retention in care, adherence, and viral load monitoring or viral suppression.
Context	Healthcare facilities and community-based service delivery settings in sub-Saharan African countries.
Study designs included	Peer-reviewed primary research employing quantitative, qualitative, or mixed-methods designs, including randomized controlled trials, quasi-experimental studies, cohort studies, case-control studies, cross-sectional studies, implementation studies, and qualitative research.
Publication types excluded	Editorials, commentaries, opinion papers, conference abstracts without full-text publications, and narrative reviews.
Eligibility framework	Population–Concept–Context (PCC) framework.

Table 2 summarises the inclusion and exclusion criteria used to determine eligibility of studies for the review.

**Stage 3: Selecting studies:** Study selection will follow the recommendations of the Joanna Briggs Institute for scoping reviews and will be reported using the PRISMA-ScR flow diagram. All retrieved records will be imported into EndNote for reference management and duplicate removal before being transferred to Rayyan application [57] to facilitate independent screening. Prior to formal screening, the review team will conduct a calibration exercise using a sample of studies to ensure consistent interpretation and application of the eligibility criteria.

Two reviewers will independently screen titles and abstracts against the predefined PCC based eligibility criteria. Studies considered potentially eligible will undergo full text assessment by the same reviewers. Any disagreements at either stage will be resolved through discussion and consensus. Where consensus can not be reached, a third reviewer will adjudicate.

For studies with multiple publications, reports will be collated and treated as a single study, with the most comprehensive and up to date publication serving as the primary reference while extracting relevant information from companion publications where necessary. The reason for excluding studies at the full text screening stage will be documented, and the complete study selection process will be presented in a PRISMA-ScR flow diagram to ensure transparency and reproducibility.

**Stage 4: Extracting data:** The Joanna Briggs Institute’s data extraction tool template will be used to extract relevant data. Key details will be gathered from the selected studies, including the publication year, country and context, study design, conceptual framework, objectives, methods, and findings.

To ensure the validity of the process, the extraction form will be piloted, tested on a few studies, and discussed with co-investigators. Two independent reviewers will then extract the data, and any disagreements will be resolved by consulting a third reviewer.

**Stage 5: Collating, summarising and reporting the outcomes: *Data collation and organization.*** The extracted data will be systematically collated in a structured manner to ensure a comprehensive synthesis of findings. Studies will be categorized based on the type of health system intervention, geographical location, study design, population characteristics, and key outcomes. Interventions will be further grouped into thematic areas such as healthcare workforce interventions, service delivery modifications, policy and governance changes, and community-based initiatives. Descriptive statistics and narrative synthesis will be used to summarize the data.

***Summarising key findings.*** The number of studies that met the inclusion criteria and were analysed will be listed. The general overview of the findings will be summarized and shared and specific interventions that improved access to HIV services will be listed and elaborated.

***Reporting the outcomes.*** The outcomes of the scoping review will be presented using tables and thematic analysis. A summary of key interventions, their mechanisms of impact, and associated HIV services access improvements among children will be provided. The evidence base will be assessed for gaps, highlighting areas where further research is needed, such as the long-term sustainability of interventions and their adaptability across different healthcare settings.

#### Status and timeline of the scoping review.

The literature search for this scoping review commenced in the last quarter of 2025. Title and abstract screening will be completed in July 2026. Full-text screening will be done between August and October 2026. Data extraction is planned for November 2026. The final results of the review are expected to be available in December 2026.

### Sub study 3: To design and develop a context-appropriate intervention package that can be used to improve access to HIV services among children in Malawi

#### Study design.

This study will employ a modified electronic Delphi (e-Delphi) approach [58] to develop a consensus based package of health system interventions to improve access to HIV services among children (0–14 years) living with HIV in Malawi. The electronic Delphi approach was selected because it enables structured consensus building among geographically dispersed experts while maintaining anonymity, reducing the influence of dominant individuals, and allowing participants to reconsider their opinions in light of controlled group feedback.

A Multidisciplinary steering committee comprising three to five members, including the principal investigator and co-investigators, will oversee the study. Members will have expertise in paediatric HIV, implementation science, health systems research, epidemiology and public health, with at least one member possessing extensive experience in HIV service delivery within Malawi. The steering committee will be responsible for refining the Delphi questionnaires, monitoring the Delphi process, reviewing qualitative feedback, and ensuring methodological rigour. Steering committee members will facilitate the process but will not participate as Delphi panellists.

Evidence generated from sub study 2 (the scoping review) will provide the initial list of candidate interventions for the modified eDelphi process. The scoping review will synthesize intervention characteristics, including intervention components, target populations, implementation settings, reported effectiveness, implementation barriers and facilitators and contextual considerations. These findings will form the basis of the round 1 questionnaire.

**Phase 1: Expert panel selection and recruitment:** A purposive maximum-variation sampling strategy [59] will be used to recruit a multidisciplinary panel representing the breadth of paediatric HIV expertise in Malawi. Eligibility criteria: Experts will be eligible if they: have at least five years` experience in paediatric HIV care, HIV programme implementation, HIV policy development, health systems strengthening, implementation research, or community based HIV programming; currently work or have worked within Malawi during the previous five years; hold positions within government, academia, implementing partners, professional associations, health facilities, or civil society organizations involved in paediatric HIV; demonstrate evidence of expertise through senior professional responsibilities, peer-reviewed publications, national HIV technical working group membership, programme leadership, or recognized technical experience; and are willing to participate in all Delphi rounds.

Individuals unable to commit to repeated rounds or whose expertise lies outside paediatric HIV or health systems strengthening will be excluded. To minimize selection bias, experts will be identified from multiple independent sources, including ministry of health (HIV) technical working group membership lists, professional associations, university departments, implementing partners, civil society organisations, and authorship of relevant peer reviewed publications. Professional networks of the investigators` will be used only to supplement these sources rather than serving as the primary recruitment strategy.

Approximately 20–30 experts will be recruited. This range is consistent with recommendations for heterogeneous Delphi panels while allowing for anticipated attrition across successive rounds [60].

Eligible experts will receive an electronic invitation containing the participant information sheet, consent form, study timeline, and estimated time commitment.

**Phase 2: Questionnaire development and pilot testing (round 1 questionnaire):** The Round 1 questionnaire will be developed directly from the interventions identified in the scoping review. Each intervention will include: a standardized description; target population; implementation setting; evidence summary; and anticipated implementation considerations. Experts will independently assess each intervention using a 5-point Likert scale for: relevance to improving access to HIV services among children in Malawi; feasibility within the Malawian health system. Open-ended questions will accompany every intervention to allow participants to: justify their ratings; identify implementation barriers and facilitators; recommend modifications; and propose additional interventions not identified during the scoping review.

Prior to administration, the questionnaire will undergo content validation by the steering committee and pilot testing with 5–8 individuals who meet the expert eligibility criteria but will not participate in the Delphi panel. The pilot will evaluate clarity, completeness, questionnaire length, usability of the electronic platform, and interpretation of rating scales. Feedback from the pilot will be incorporated before Round 1 is launched.

**Phase 3: Delphi consensus rounds:** The Delphi process will be conducted electronically using a secure online survey platform that preserves participant anonymity throughout all rounds. Each round will remain open for two to three weeks, with reminder emails sent after one and two weeks.

***Round 1.*** Experts will independently rate all interventions. Quantitative analysis will include: median scores; interquartile ranges (IQR); and percentage agreement. Qualitative responses will undergo thematic content analysis to identify suggested modifications, implementation concerns, and newly proposed interventions.The steering committee will revise intervention wording where clarification is required and incorporate newly proposed interventions that align with the study objectives.

***Round 2.*** Each participant will receive individualized feedback comprising: their previous rating; the panel median; the interquartile range; percentage agreement; and an anonymized summary of panel comments. Experts will then reconsider and re-rate each intervention. New interventions generated during Round 1 will be introduced for initial rating.

***Round 3.*** Only interventions failing to achieve consensus during Round 2 will be re-rated. Participants will again receive anonymized statistical summaries and qualitative feedback before providing their final ratings.

***Consensus definition:*** Consensus will be defined a priori using all of the following criteria: median rating ≥4 for both relevance and feasibility; interquartile range (IQR) ≤1; and at least 75% of participants assigning ratings of 4 or 5. Interventions meeting all three criteria will be considered to have achieved consensus for inclusion. Interventions failing to satisfy these criteria after the final Delphi round will be classified as having no consensus and will not be included automatically in the final intervention package.

***Stopping rule:*** The Delphi process will conclude when one of the following occurs: all interventions have reached either consensus or non-consensus classification; three Delphi rounds have been completed; or the proportion of interventions changing consensus status between consecutive rounds falls below 10%, indicating response stability. No more than three rounds are anticipated because additional rounds generally provide limited improvements while increasing participant fatigue.

**Phase 4: Development of the intervention package:** Interventions reaching consensus will be synthesized into a comprehensive package organized according to: level of health service delivery (community, primary care, district hospital, tertiary care); stage of the paediatric HIV cascade (testing, linkage, ART initiation, retention, adherence, viral suppression); and health system building blocks where appropriate.

Each intervention will include: implementation rationale; expected mechanism of action; implementation requirements; contextual facilitators; anticipated barriers; recommendations for adaptation within Malawi. A conceptual implementation framework describing integration within existing HIV services will also be developed.

**Stakeholder dissemination and validation:** Following completion of the Delphi process, a separate stakeholder dissemination workshop will be convened with policymakers, programme managers, implementing partners, civil society representatives, and interested Delphi panelists. This workshop will not constitute an additional Delphi round and will not modify the consensus findings. Rather, its purpose will be to discuss implementation considerations, identify resource and policy implications, and enhance stakeholder ownership of the intervention package. By separating dissemination from consensus generation, participant anonymity and the methodological independence of the Delphi process will be preserved.

#### Reporting.

The study will be reported in accordance with the CREDES reporting recommendations for Delphi studies [61]. Recruitment, participant characteristics, response rates, attrition, questionnaire modifications, consensus outcomes, and reasons for non-consensus will be documented transparently. Both quantitative consensus statistics and qualitative findings will be presented to support the final intervention package.

#### Timeline for this sub study.

Participants recruitment is scheduled for December 2026, followed by data collection in the next three months. Final findings of this sub study would be available in June 2027.

### Sub study 4: Assessing possible feasibility and acceptability of the proposed package of interventions to improve access to HIV services among children at Kabudula rural hospital, Lilongwe district, Malawi

#### Study design.

This sub study will employ a convergent mixed-methods case study design to assess the perceived feasibility and acceptability of the intervention package developed in sub study number three. A convergent design is appropriate because quantitative and qualitative data will be collected during the same study period, analysed separately, and integrated during interpretation to provide a comprehensive understanding of implementation outcomes. The intervention package will be presented through standardised three to seven-minute video scenarios demonstrating how the proposed interventions could be implemented within routine paediatric HIV services delivery points. The videos will ensure that all participants receive identical information before data collection, thereby reducing variability in interpretation of the intervention package.

The quantitative component will measure perceived acceptability, appropriateness and feasibility using validated implementation outcome measures adapted from the Acceptability of Intervention Measure (AIM), Intervention Appropriateness Measure (IAM), and Feasibility of Intervention Measure (FIM) [62].

The qualitative component will explore participants’ explanations for their ratings, contextual implementation barriers and facilitators, and recommendations for improving the intervention package.

Integration of quantitative and qualitative findings will occur during interpretation using joint displays and narrative triangulation. Quantitative findings will identify the level of perceived implementation outcomes, while qualitative findings will explain the reasons underlying these perceptions and provide contextual insights to refine the intervention package.

#### Study setting.

The study will be conducted at Kabudula community hospital in Lilongwe district, Malawi. The hospital was purposively selected because it provides comprehensive children HIV services across both facility and community platforms, including collaboration with community health workers. The hospital serves a predominantly rural population facing many of the barriers to paediatric HIV service access identified in previous sub studies including long travel distances, socioeconomic constraints and health workforce shortages.

As an information-rich case, the site provides an appropriate setting for an in-depth feasibility assessment before wider implementation.

Although findings may not be statistically generalisable to all Malawian settings, the purpose of this case study is to generate contextually relevant implementation evidence and identify practical considerations that can inform future multi-site evaluation.

#### Study population.

Three stakeholder groups will participate: Healthcare workers involved in paediatric HIV care, community healthcare workers supporting HIV service delivery, caregivers of children aged 0–14 years receiving HIV services.

To improve the relevance of the intervention scenarios, caregivers will be stratified according to children’s developmental stages (0–4, 5–9 and 10–14 years). Separate video modules will therefore be developed for each caregiver group, while distinct modules will be developed for facility-based healthcare workers and community healthcare workers.

#### Sampling strategy.

A purposive maximum-variation sampling will be used to ensure representation of diverse experiences across participant groups [59].

#### Sample size.

**Quantitative component:** The quantitative survey is intended to estimate perceived implementation outcomes rather than test intervention effectiveness. Consequently, sample size has been guided by recommendations for implementation feasibility studies rather than formal hypothesis testing.

Approximately: 20 healthcare workers, 20 community healthcare workers and 30 caregivers (10 per child age category) will complete the AIM/IAM/FIM questionnaire.

This sample is considered sufficient to provide stable descriptive estimates of implementation outcomes across stakeholder groups while remaining feasible within a single-site implementation assessment.

**Qualitative component:** Qualitative sample sizes have been guided by the principle of information power, whereby richer information from participants with direct experience requires fewer participants. Approximately: 15–20 individual interviews with healthcare workers, 15–20 individual interviews with community healthcare workers, three focus group discussions comprising 6–8 caregivers each will be conducted.

Recruitment will continue until thematic saturation is achieved, defined as the point at which no substantively new themes emerge from successive interviews [63].

#### Participants recruitment.

Healthcare workers will receive study information during routine morning handover meetings. Recruitment will occur subsequently through individual invitations to minimise perceived pressure to participate.

Caregivers will receive general information during routine clinic health education sessions. Interested caregivers will subsequently contact the research team independently or meet study staff after clinic activities. Focus groups will be scheduled on dates separate from routine clinic appointments to minimise perceptions that participation could influence clinical care.

#### Data collection procedures.

Participants will first watch the standardised intervention video relevant to their stakeholder group. Immediately afterwards, participants will complete the quantitative AIM/IAM/FIM questionnaire. Following questionnaire completion: healthcare workers and community healthcare workers will participate in semi-structured individual interviews; caregivers will participate in focus group discussions. Collecting questionnaire responses before qualitative interviews will reduce the influence of group discussion on participants’ initial implementation ratings. Interview guides will be informed by implementation science frameworks and will explore: acceptability; feasibility; contextual appropriateness; implementation barriers; implementation facilitators; and recommendations for improving the intervention package.

All interviews will be audio-recorded with consent and supplemented by field notes documenting contextual observations.

#### Development and pilot testing of data collection tools.

Questionnaires and interview guides will be informed by findings from Sub-studies 2 and 3 together with established implementation outcome frameworks. All instruments will undergo: expert review for content validity; translation into Chichewa and independent back-translation; pilot testing among participants with similar characteristics who will not participate in the main study. Pilot testing will assess clarity, comprehension, cultural appropriateness, interview duration and questionnaire usability. Necessary revisions will be made before data collection begins.

#### Strategies to minimise bias.

Several measures will be implemented to reduce potential sources of bias.

**Social desirability bias:** Because participants are evaluating interventions developed through this research, several safeguards will be implemented. Participants will be assured that there are no correct or incorrect answers. Questionnaires will be completed anonymously using participant identification numbers rather than names. Participants will be informed that their responses will not influence their employment or access to healthcare services. Interviews will emphasize both positive and negative experiences, with interviewers actively encouraging criticism and suggestions for improvement. Healthcare workers and caregivers will be interviewed separately to minimise hierarchical influences.

**Researcher bias:** To reduce interviewer bias and enhance trustworthiness: trained independent qualitative research assistants, rather than the principal investigator alone, will conduct interviews and focus group discussions after receiving standardised training; interviewers will follow a structured interview guide; transcripts will be independently coded by at least two researchers; discrepancies will be resolved through discussion and consensus; an audit trail documenting coding decisions will be maintained; reflexive memos will be recorded throughout data collection and analysis. The principal investigator will oversee quality assurance but will not serve as the sole interviewer or coder.

#### Data analysis.

**Quantitative analysis:** Questionnaire responses will be analysed using descriptive statistics. Median scores, interquartile ranges and percentages of participants agreeing or strongly agreeing with each AIM, IAM and FIM item will be calculated separately for each stakeholder group.

Internal consistency of each implementation measure will be assessed using Cronbach’s alpha [64].

**Qualitative analysis:** Interview and focus group transcripts will undergo reflexive thematic analysis. Two researchers will independently code transcripts before jointly developing a coding framework. Themes describing implementation barriers, facilitators and recommendations will then be generated.

**Mixed-methods integration:** Integration will occur after completion of the separate quantitative and qualitative analyses.

A joint display matrix will be used to compare implementation outcome scores with qualitative themes explaining those scores. Areas of convergence, complementarity and divergence will be identified to develop an integrated interpretation of intervention feasibility and acceptability. The integrated findings will inform refinement of the intervention package prior to future implementation studies.

### Timeline for this sub study

Participants recruitment shall start in July 2027. Data collection will start in the same month and extend to the next three to four months. Results are expected to be available towards the end of 2027.

### Conceptual framework

The Socio-Ecological Model (SEM) [65] is the conceptual framework guiding this study. It draws from the HIV care cascade to identify and integrate evidence-based interventions that influence paediatric HIV service uptake across multiple levels of influence. This framework acknowledges that improving HIV services uptake among children requires coordinated interventions addressing individual, interpersonal, community, institutional, and policy-level barriers.

HIV care cascade framework guides the focus of interventions across critical stages namely HIV testing and diagnosis, linkage to care, initiation of ART, retention in care, and viral suppression.

Interventions at individual and interpersonal level focus on improving knowledge, motivation, and behaviour among children and their caregivers, for example peer support for caregivers [66], SMS reminders for appointments/adherence [67], school-based HIV education and testing [68] and cash transfers to improve caregiver capacity and access [69]. Interventions at community level will address stigma, social norms, and community engagement, examples are community health worker follow-up and psychosocial support [70], and home-based HIV counselling and testing [71].

Interventions at institutional/health system level will focus on improving service delivery, access, and quality of care, for example point of care early infant diagnosis (POC-EID) [72], family centred index case testing [73], service integration into routine child health/immunization clinics [74,75], and task shifting to trained lay health workers [76].

Interventions at policy and structural level will create enabling environments through supportive policies, funding, and infrastructure, examples are supportive national paediatric HIV strategies, harmonized guidelines for integration and task-shifting and investment in health information systems and digital tools.

The interventions, when packaged and implemented together, are hypothesized to increase early identification of HIV-positive children (via HBHCT, family testing, and school outreach), strengthen linkage to care (through CHW support, SMS reminders), accelerate ART initiation (via POC-EID and integration into routine care), improve retention and adherence (through caregiver support, CHWs, cash transfers) and ultimately improve health outcomes and achieve higher rates of viral suppression in children.

### Ethical considerations

Ethical approval for the overall study will be obtained from the Health Research Ethics Committee of Stellenbosch University and the Malawi National Health Sciences Research Ethics Committee (NHSRC). Administrative permission will also be obtained from the Malawi Ministry of Health’s Department of HIV and AIDS.

Sub-study 1 analysed secondary, fully anonymised national HIV programme data containing no personally identifiable information and involving no direct participant contact. Individual informed consent was therefore not required. Data are stored on encrypted, password-protected systems accessible only to authorised study personnel and used solely for research purposes. Findings were reported in aggregate to ensure confidentiality.

Sub-study 2 is a scoping review based exclusively on published, de-identified literature and does not involve human participants. Consequently, individual informed consent is not required. Institutional ethical approval will nevertheless be obtained in accordance with Stellenbosch University and NHSREC requirements.

Sub-studies 3 and 4 involve human participants and will be conducted only after obtaining ethical approval from both ethics committees. Written informed consent will be obtained from all participants before data collection. Participants will receive information about the study objectives, procedures, potential risks and benefits, confidentiality measures, and their right to decline participation or withdraw at any time without consequence. All data will be anonymised, with personal identifiers removed from transcripts and study records, and interviews and focus group discussions will be conducted in private settings to safeguard confidentiality. The study presents minimal risk to participants, while the anticipated benefits include contributing evidence to strengthen access to paediatric HIV services in Malawi.

## Discussion

This study is expected to generate evidence to inform strategies for improving access to HIV services among children in Malawi. The first sub-study assessed national progress towards the UNAIDS 95-95-95 targets and identified disparities across the HIV care continuum.

Although overall programme performance demonstrated substantial progress, disaggregated analyses revealed persistent inequities, particularly among children aged 0–14 years. Identifying these gaps will help prioritise populations requiring targeted interventions and guide equitable programme strengthening.

The second sub-study will synthesise evidence on health system interventions implemented to improve paediatric HIV service access in sub-Saharan Africa. By mapping intervention characteristics, implementation approaches, and reported outcomes, the review will identify strategies with potential applicability to Malawi while highlighting important evidence gaps requiring further investigation.

The third and fourth sub-studies will translate these findings into practice through the development and assessment of a context-specific intervention package. Integrating evidence from national programme data, the scoping review, and stakeholder consensus will ensure that the proposed package is evidence-informed and tailored to the Malawian health system. Assessing its possible feasibility and acceptability will provide valuable implementation insights to inform future piloting and scale-up. Collectively, the four sub-studies will generate complementary evidence to support the design of effective, contextually appropriate strategies for strengthening paediatric HIV service delivery in Malawi.

### Strengths and limitations of the study

A major strength of this study is its integrated multi-methods design, which combines national programme data analysis, evidence synthesis, and stakeholder-informed intervention development. This sequential approach enables the study to move beyond describing service gaps to identifying evidence-based solutions and assessing their implementation potential. The intervention package will be informed by empirical national data, regional evidence, and expert consensus, enhancing its relevance, feasibility, and potential for policy translation within the Malawian context.

Several limitations should be acknowledged. The first sub-study relies on routinely collected aggregate programme data, which may contain inaccuracies, missing values, or reporting inconsistencies. These limitations were mitigated by using validated national datasets and conducting data quality checks before analysis.

The scoping review is restricted to studies conducted in sub-Saharan Africa and published in English. While this may exclude relevant evidence from other low- and middle-income countries and non-English publications, the review is designed to maximise contextual relevance to Malawi.

The Delphi process may be affected by challenges in recruiting and retaining busy experts and by the potential for facilitator influence during consensus development. These risks will be minimised through careful participant engagement, structured communication, anonymous survey rounds, and standardised presentation of feedback to preserve neutrality throughout the consensus process.

## Conclusion

Despite substantial progress in HIV prevention, treatment, and care, children in Malawi continue to experience significant gaps across the HIV care continuum, limiting progress toward the UNAIDS 95–95–95 targets and the goal of ending AIDS as a public health threat. Addressing these inequities requires robust evidence on existing service delivery gaps and effective, contextually appropriate strategies to improve access to HIV services for children.

This multifaceted study combines analysis of national HIV programme data, a scoping review of health system interventions in sub-Saharan Africa, and the co-design and evaluation of a context-specific intervention package for Malawi. By generating evidence on paediatric HIV programme performance, synthesizing effective health system interventions, and assessing their feasibility and acceptability within the Malawian context, the study will provide actionable evidence to strengthen paediatric HIV service delivery. The findings are expected to inform policy and programme implementation, improve access to HIV services for children, and support Malawi`s progress toward sustained epidemic control.
