# Supplementary material for: Investigating strategies to enhance access to HIV services among children in Malawi: A multifaceted research approach, study protocol
Source: PLoS One. 2026 Sep 3;21(9):e0357408. doi: 10.1371/journal.pone.0357408 (PMC13541177; doi:10.1371/journal.pone.0357408)
Supplement: Supplementary file 1 [file pone.0357408.s002.docx]

**COVER LETTER**

8^th^ July 2026

**RE: REVISED MANUSCRIPT SUBMISSION TO PLOS ONE**

# **Manuscript Title:** Investigating strategies to enhance access to HIV services among children in Malawi: A multifaceted research approach, study protocol

Dear Editor,

On behalf of my co-authors, I am pleased to submit the revised version of our manuscript entitled **"** **Investigating strategies to enhance access to HIV services among children in Malawi: A multifaceted research approach, study protocol "** for further consideration for publication in *PLOS ONE*.

We thank you and the reviewers for the thorough and constructive evaluation of our manuscript. We greatly appreciate the insightful comments, which have substantially strengthened the scientific rigor, methodological transparency, and overall clarity of the protocol. We have carefully considered every comment raised by the reviewers and have revised the manuscript accordingly. A detailed point-by-point response to all reviewer comments accompanies this submission.

The major revisions undertaken include, but are not limited to, the following:

- The study rationale and research question have been refined by clearly defining the concepts of "access to HIV services" and "approaches," strengthening the justification for the evidence gap, and articulating the conceptual linkage between the four sub-studies through a sequential mixed-methods framework supported by a workflow figure.
- The manuscript has been revised to clearly distinguish completed work from prospective study components, thereby resolving ambiguities regarding the protocol status.
- Methodological rigor has been strengthened across all study components through expanded descriptions of study variables, outcome definitions, statistical analyses, data integration procedures, sampling strategies, instrument development, consensus criteria, and bias mitigation measures.
- The scoping review methodology has been aligned with current Joanna Briggs Institute guidance and the PRISMA Extension for Scoping Reviews (PRISMA-ScR), with consistent application of the Population–Concept–Context framework and clarification of eligibility criteria and search strategy.
- The modified Delphi methodology has been expanded to include explicit expert eligibility criteria, consensus definitions, stopping rules, questionnaire development procedures, and clarification of the role of the post-Delphi stakeholder workshop.
- The feasibility component has been reframed to evaluate anticipated feasibility, acceptability, and appropriateness rather than real-world implementation feasibility, with additional detail provided regarding sampling, recruitment, mixed-methods analysis, and study limitations.
- Additional information has been incorporated regarding data management, ethical considerations, methodological transparency, and study limitations to improve reproducibility and compliance with reporting standards.
- The manuscript has undergone comprehensive language editing to improve clarity, consistency, grammar, terminology, and formatting throughout.

In response to the journal's editorial requirements, we also wish to clarify the following:

1. **PLOS ONE formatting requirements**

We confirm that the manuscript has been revised to comply with the *PLOS ONE* formatting guidelines, including formatting of the title page, manuscript structure, headings, tables, figures, references, and file naming conventions in accordance with the journal's style templates.

2. **Competing Interests**

The authors declare that no competing interests exist.

3. **Data Availability Statement**

As this manuscript describes a study protocol, no datasets have yet been generated or analysed.

The revised manuscript now includes the following Data Availability Statement:

"As this manuscript describes a study protocol, no datasets have yet been generated or analysed. Upon completion of the study, de-identified quantitative datasets supporting the published findings will be deposited in an appropriate public data repository where permitted by national data-sharing agreements. Qualitative data will be shared in a de-identified form upon reasonable request, subject to ethical approval, participant confidentiality, and Malawi Ministry of Health data governance requirements. Secondary national programme data used in this study remain subject to Ministry of Health data access policies and cannot be shared by the authors without permission from the data custodian."

4. **Ethics Statement**

We confirm that the ethics statement now appears only within the Methods section of the manuscript, in accordance with *PLOS ONE* requirements, and has been removed from all other sections.

5. **Reviewer-recommended references**

We carefully evaluated all publications suggested by the reviewers. Relevant methodological and substantive references have been incorporated where appropriate to strengthen the scientific justification and reporting of the study. References that were not directly relevant to the study objectives or methodology were not included.

We believe that the revisions have substantially strengthened the manuscript and have addressed all concerns raised by both the reviewers and the editorial office. We hope that the revised manuscript is now suitable for publication in *PLOS ONE*.

Thank you for your time and consideration. We look forward to your favorable response.

Yours sincerely,

**Dr. Bilaal Wilson Matola**
Principal Disease Control Officer
HIV Department
Ministry of Health, Malawi

On behalf of all co-authors

| **No** | **Reviewers’ comment (s)** | **Response by the researchers** | **Location (Page Number, Section)** |
| --- | --- | --- | --- |
| **Responses to Reviewer #1** | | | |
| 1. | 1. Study rationale and research question  The manuscript addresses a valid and important academic and public health problem. The disparity between adult and paediatric HIV outcomes in Malawi provides a strong rationale for the study .  However, the research question remains too broad and insufficiently operationalized. Terms such as “access” and “approaches” are not clearly defined, making the precise scope of inquiry unclear. It is not explicit whether the study focuses primarily on HIV testing, diagnosis, ART initiation, retention in care, viral suppression, health systems strengthening, or implementation strategies.  The manuscript would also benefit from a stronger justification for the claimed evidence gap, particularly the assertion that there is limited systematic evidence on effective interventions in this area.  In addition, the rationale for combining four distinct methodological studies into a single protocol is not sufficiently articulated. While the individual components are understandable, the manuscript does not clearly explain the overarching logic linking:  * national epidemiological analysis, * evidence synthesis, * Delphi consensus development, * and feasibility assessment.  A clear conceptual workflow or logic model is needed. | The Introduction has been substantially revised to more precisely define the key constructs investigated in this study. Specifically, "access to HIV services" is now explicitly defined to include the continuum of pediatric HIV services comprising HIV testing, diagnosis, linkage to care, antiretroviral therapy (ART) initiation, retention in care, viral load monitoring, viral suppression, and transition from pediatric to adult HIV services. Similarly, the term "approaches" has been clarified to refer to evidence-informed service delivery models, implementation strategies, and health system interventions designed to improve access and continuity of HIV care.  To strengthen the justification for the evidence gap, additional literature has been incorporated demonstrating that although pediatric HIV outcomes have improved globally, there remains limited synthesized evidence regarding effective transition models and integrated implementation strategies for children and adolescents living with HIV in low-resource settings.  The rationale for integrating the four sub-studies has also been expanded. We now explicitly describe the sequential mixed-methods design whereby:   1. national epidemiological analysis identifies service delivery gaps; 2. the scoping review synthesizes global evidence on interventions addressing those gaps; 3. the modified Delphi process contextualizes and prioritizes evidence through stakeholder consensus; and 4. the feasibility assessment evaluates the acceptability and implementation potential of the proposed transition framework. | Introduction, Page 4 - 8 |
| 2. | 2. Protocol coherence and study status  A major concern is inconsistency regarding the protocol’s status.  This manuscript is presented as a prospective study protocol, yet:  * Sub-study 1 is described as already completed and published. * Sub-study 2 appears partially completed. * Other components remain prospective.  This creates substantial ambiguity regarding whether this is:  * a true protocol, * a hybrid progress report, * or a doctoral programme methods overview.  Completed work should not be presented as future planned research without clear explanation. | The manuscript has been revised to clearly distinguish completed work from planned activities. Sub-study 1 represents previously completed work that provides the epidemiological foundation for the subsequent phases of the research programme. It is now explicitly described as preliminary evidence informing the development of the remaining prospective components rather than as future work. Similarly, the current status of each sub-study is clearly indicated throughout the manuscript. This clarification aligns the protocol with recommendations for reporting complex, multi-phase research programmes. | Methodology Page 9 - 11 |
| 3. | 3. Technical soundness and methodological rigor  The protocol includes substantial methodological description, but important weaknesses reduce confidence in its technical robustness.  Key concerns:  * insufficient detail regarding analytic decision-making; * unclear outcome definitions; * limited explanation of data integration procedures; * absence of clear distinction between confirmatory and exploratory analyses; * inadequate justification for some design choices.  The protocol appears conceptually ambitious but methodologically uneven. | Throughout the manuscript we have expanded the methodological descriptions by:   - clearly defining primary and secondary outcomes; - specifying confirmatory and exploratory analyses where applicable; - describing procedures for integrating findings across the four sub-studies using a sequential evidence synthesis framework; - providing additional justification for methodological choices; and - strengthening descriptions of data management and analysis. | Introduction  Page 4 – 8  Methodology  Page 8 - 27 |
| 4. | 4. Sub-study 1: Secondary quantitative analysis  This component raises several concerns.  First, because this study is already described as completed and published, it should not be framed as prospective protocol work.  Second, the statistical analysis plan lacks sufficient rigor. The manuscript proposes descriptive analyses and proportion comparisons , but the dataset spans more than a decade, suggesting the need for stronger trend analysis methods.  Third, key methodological details are missing:  * precise variable definitions; * unit of analysis; * treatment of missing data; * reconciliation between DHAMIS programme data and Spectrum estimates; * assumptions underlying denominator construction.  Without these details, reproducibility is limited. | The manuscript has been revised to clarify that Sub-study 1 has already been completed and serves as the evidence base informing subsequent phases.  Additional methodological detail has been added, including:   - operational definitions of all study variables; - unit of analysis; - treatment of missing data; - assumptions used in denominator construction; - procedures used to reconcile routine programme data with Spectrum estimates.   Regarding trend analysis, we agree that longitudinal datasets warrant more robust analytical approaches. Accordingly, we have revised the statistical analysis plan to include temporal trend analyses using regression-based methods where appropriate, rather than relying solely on descriptive comparisons. | Methodology  Page 9 - 11 |
| 5. | 5. Sub-study 2: Scoping review  The scoping review methodology is broadly structured but contains methodological inconsistencies.  Concerns include:  * mixing multiple frameworks (Arksey & O’Malley, Levac, JBI, PRISMA-P, PRISMA-ScR) without clear methodological coherence; * inappropriate inclusion of formal risk-of-bias tools, which is not standard for scoping reviews unless explicitly justified; * inconsistency between PCC and PICO frameworks; * mismatch between stated target population (children 0–14 years) and inclusion of adolescents in search terms; * unsupported dismissal of language bias resulting from English-only inclusion.  This component requires methodological tightening. | The methodology has been revised to align explicitly with the JBI Manual for Evidence Synthesis, while reporting will follow PRISMA-ScR. Earlier versions cited multiple methodological frameworks because JBI methodology builds upon the foundational work of Arksey and O'Malley and incorporates subsequent refinements by Levac et al. To improve clarity, we now explicitly state the primary methodological framework and explain the role of supporting guidance.  The protocol has also been revised to:   - use the PCC framework consistently throughout; - remove the formal risk-of-bias assessment, as this is generally not required for scoping reviews; - clarify the target population and corresponding search strategy; - acknowledge that restricting inclusion to English-language publications may introduce language bias rather than suggesting that such bias is absent. | Methodology  Page 12 - 15 |
| 6. | 6. Sub-study 3: Modified e-Delphi process  The Delphi methodology requires greater precision.  Specific concerns:  * unclear expert eligibility criteria; * reliance on professional networks, creating potential selection bias; * ambiguous consensus definitions; * no clearly defined stopping rule for Delphi rounds; * insufficient detail on questionnaire development and piloting; * risk that the planned in-person workshop compromises Delphi anonymity and independence.  These methodological details should be clarified to improve rigor and reproducibility. | The Delphi methodology has been substantially strengthened.  The revised manuscript now specifies:   - explicit expert eligibility criteria; - purposive sampling procedures and strategies to maximize diversity while minimizing selection bias; - predefined consensus thresholds; - stopping criteria for Delphi rounds; - questionnaire development and pilot testing procedures.   We also clarify that the face-to-face workshop will occur only after completion of the anonymous Delphi rounds and will be used solely to discuss implementation considerations rather than to establish consensus, thereby preserving the independence of Delphi responses. | Methodology  Page 17 - 21 |
| 7. | 7. Sub-study 4: Feasibility and acceptability assessment  This is the weakest methodological component.  The proposed design assesses participant perceptions after watching a short video illustrating interventions rather than assessing interventions under real implementation conditions .  This raises major validity concerns.  Watching a simulated intervention does not constitute genuine feasibility testing. Participants cannot reliably assess:  * operational feasibility, * implementation burden, * workflow integration, * sustainability, * staffing implications.  At best, this design assesses perceived acceptability, appropriateness, or hypothetical feasibility. | We agree that the proposed activity is more appropriately characterized as an assessment of anticipated feasibility, acceptability, and appropriateness, rather than a full implementation feasibility study. The manuscript has therefore been revised accordingly.  The objectives have been reframed to emphasize that participants will evaluate the perceived acceptability, appropriateness, usability, and anticipated implementation challenges of the proposed framework after reviewing the demonstration materials.  Additional methodological details have also been incorporated, including:   - sample size justification based on feasibility study recommendations; - recruitment procedures; - quantitative and qualitative analytic approaches; - strategies to reduce social desirability bias; - investigator triangulation during qualitative analysis.   We also acknowledge in the limitations that operational feasibility under real-world implementation conditions will require subsequent implementation research. | Methodology  Page 22 – 27 |
| 8 | 8. Replicability and methodological transparency  The manuscript provides moderate methodological detail, but reproducibility remains limited.  Missing or insufficiently described elements include:  * sample size calculations or power analysis for quantitative work; * justification for participant numbers in Delphi and feasibility studies; * instrument validation procedures; * data handling procedures; * bias mitigation strategies; * integration methods for mixed-methods findings.  More methodological precision is needed for another researcher to replicate the work. | We agree that additional methodological detail strengthens reproducibility.  The revised manuscript now includes:   - sample size justification for feasibility and Delphi studies; - justification for participant numbers; - instrument adaptation procedures; - data management procedures; - bias mitigation strategies; - explicit mixed-methods integration procedures.   We acknowledge that formal power calculations are not applicable to the feasibility assessment because its primary objective is estimating implementation parameters rather than hypothesis testing. | Methodology  Page 8 – 27 |
| 9. | 9. Data availability  The manuscript does not adequately meet PLOS ONE data-sharing expectations.  Although this is a protocol manuscript, the authors should clearly specify how data generated from the study will be shared upon completion.  Current concerns:  * no named public repository; * no description of what datasets will be shared; * no clarification of access restrictions for national programme data; * no plan for sharing de-identified qualitative data.  A PLOS-compliant data availability statement is required. | The Data Availability Statement has been revised to comply with PLOS ONE requirements.  The revised statement specifies:   - which datasets will be generated; - that de-identified quantitative and qualitative datasets supporting published findings will be deposited in an appropriate public repository (or made available through a controlled-access repository if required by data-sharing agreements); - that access to national programme data remains subject to Ministry of Health approval.   This approach balances transparency with participant confidentiality and data governance requirements. | Methodology  Page 11 |
| 10. | 10. Ethics  Ethics reporting is inconsistent.  The manuscript states ethical approval “will be obtained,” while submission metadata suggests approval already exists.  Additional concerns:  * ambiguity regarding secondary data ethics approval versus exemption; * confidentiality risks associated with recruitment through HIV clinic settings; * no explicit governance for use of participant actors in intervention videos.  These issues should be clarified. | The ethics section has been revised to eliminate inconsistencies regarding approval status. The manuscript now clearly distinguishes approvals already obtained from approvals required for forthcoming study components. We also clarify:   - governance arrangements for secondary programme data; - procedures to protect confidentiality during participant recruitment; - consent procedures for actors participating in the demonstration videos.   These revisions align the manuscript with international ethical reporting recommendations. | Ethical considerations  Page 28 – 29 |
| 11. | 11. Language, presentation, and clarity  The manuscript is generally understandable, but substantial editing is required before publication consideration.  Concerns:  * grammatical and typographical errors; * awkward sentence construction; * excessive repetition; * inconsistent terminology; * formatting inconsistencies;  The manuscript would benefit from thorough language editing. | The manuscript has undergone comprehensive language editing to improve grammar, sentence structure, consistency of terminology, formatting, and readability. Repetitive text has been removed, terminology has been standardized throughout, speculative language has been eliminated from protocol sections, and the limitations section has been expanded. | All manuscript sections  Page 2 – 31 |
|  | Minor comments  * Define DHAMIS at first use. * Clarify whether adolescents are included. * Justify selection of Kabudula Community Hospital. * Clarify whether adapted AIM/IAM/FIM tools retain validation properties. * Standardize terminology throughout. * Improve formatting consistency. * Remove speculative findings language from protocol sections. * Strengthen limitations discussion. * Provide clearer summary workflow figure. | - DHAMIS is now defined at first mention. - The inclusion of adolescents has been clarified throughout the manuscript using standardized WHO age definitions. - The rationale for selecting Kabudula Community Hospital has been expanded to explain its relevance as the feasibility assessment site. - Additional information has been included regarding the adaptation and use of the Acceptability of Intervention Measure (AIM), Intervention Appropriateness Measure (IAM), and Feasibility of Intervention Measure (FIM), including evidence supporting the validity of adapted implementation outcome measures following contextual modification and pilot testing. - Terminology has been standardized throughout the manuscript. - Formatting inconsistencies have been corrected. - Speculative statements have been removed from protocol sections. - The limitations section has been strengthened. - A study workflow figure illustrating the sequential design has been added. | All manuscript sections  Page 2 – 31 |
| **Responses to Reviewer #2** | | | |
| 1. | The protocol is sound, has clear rationale, multi-method design combining secondary data review and literature review, is aligned with global target, has mentioned clear approval, consent and confidentiality demonstrated. It's based on Malawi with a possibility of it being scaled. | No changes were required in response to this comment | Not applicable |
| 2. | There is need to condense the abstract. | The abstract has been revised to improve conciseness while ensuring that all essential elements recommended for protocol reporting are retained. Redundant background information has been removed, and the objectives, methodology, expected outcomes, and significance have been presented more succinctly in accordance with recommendations for protocol reporting. | Abstract  Page 2 |
| 3. | Could also mention the existing strategies which have failed if any | The Introduction has been expanded to briefly describe existing approaches implemented in sub-Saharan Africa and globally. | Introduction  Page 4 – 8 |
| 4. | Sub-study 1: Justification for choice of tests (Chi-square versus Fisher's Exact test) could be clearer. | The statistical analysis section has been revised to explicitly justify the selection of statistical tests. We now state that the Chi-square test will be used to compare categorical variables when expected cell frequencies meet the underlying assumptions (expected frequency ≥5 in at least 80% of cells). Fisher's Exact test will instead be applied when expected frequencies are small, ensuring valid inference despite sparse data. This approach follows established recommendations for categorical data analysis. | Methodology  Page 10 – 11 |
| 5. | Sub-study 2: Scoping review lacks detail on inclusion/exclusion criteria, databases, and search strategy. | The scoping review methodology has been expanded to align more closely with current methodological guidance. Specifically, we have now:   - Clearly defined the inclusion and exclusion criteria using the Population–Concept–Context (PCC) framework. - Specified the electronic databases to be searched (PubMed/MEDLINE, Embase, Scopus, Web of Science, and Google Scholar), together with relevant grey literature sources. - Added a description of the search strategy, including the use of controlled vocabulary (e.g., MeSH terms), free-text keywords, Boolean operators, and adaptation of search strings across databases. - Indicated that study selection and reporting will follow the PRISMA-ScR reporting guideline.   These revisions improve transparency and reproducibility. | Methodology  Page 12 – 15 |
| 6. | Sub-study 3: The modified e-Delphi process is appropriate, but criteria for expert selection should be specified. | The manuscript has been revised to explicitly define the expert selection criteria. Experts will be purposively selected based on demonstrated experience in adolescent HIV care, transition programming, implementation research, HIV policy development, or clinical management. Participants will include clinicians, researchers, programme managers, Ministry of Health representatives, implementing partners, and adolescent representatives. Eligibility criteria include a minimum number of years of relevant experience and active involvement in HIV service delivery or research. These additions improve the transparency and credibility of the consensus process. | Methodology  Page 17 – 21 |
| 7. | Sub-study 4: Feasibility assessment mentions video demonstration and questionnaires, but lacks detail on sample size, recruitment, and analytic approach. | Additional methodological detail has been incorporated into the feasibility assessment. Specifically, the revised manuscript now describes:   - the target sample size and justification based on recommendations for feasibility and pilot studies rather than hypothesis testing; - participant recruitment procedures, including eligibility criteria and sampling approach; - quantitative analysis using descriptive statistics to summarize feasibility outcomes (e.g., acceptability, usability, completeness, and satisfaction); and - qualitative analysis using thematic analysis of participant feedback to identify implementation barriers and facilitators.   These additions provide greater methodological clarity while remaining consistent with the exploratory objectives of a feasibility study. | Methodology  Page 22 – 27 |
| 8. | Data availability written as N/A since it is a protocol but may give a statement on how data will be shared. | We have revised the Data Availability Statement to clarify how study data will be managed upon completion of the project. The statement now reads:  *As this manuscript describes a study protocol, no datasets have yet been generated or analysed. Upon completion of the study, de-identified datasets generated and/or analysed during the study will be made available from the corresponding author upon reasonable request, subject to institutional ethical approvals, participant confidentiality, and applicable data-sharing agreements.*  This revision aligns with current recommendations promoting transparency and responsible data sharing. |  |
| **Responses to reviewer #3** | | | |
| 1. | For Aim 1, in line 197, what informed the decision to disaggregate data into 15 and below and above 15 years old? Does adult age begin at 15? I'd recommend using WHO standardized ages and age boundaries that are less wide for a more granular comparison. | The age categorization reflects the reporting structure used within the Malawi national HIV programme and electronic medical record system. We agree, however, that internationally recognized age classifications provide greater comparability across studies. | Methodology  Page 10 – 11 |
| 2. | In line 203, why is the study team using both SPSS and Stata? I'd recommend sticking with one tool to ease collaboration among the analysts within the team. | We agree that using a single statistical software package enhances reproducibility, facilitates quality assurance, and promotes consistency in data management and analysis among team members. Consequently, the manuscript has been revised to specify that all quantitative analyses will be conducted using a single statistical software package (Stata version 15.1), which is routinely used by the research team for epidemiological analyses. | Methodology  Page 10 |
| 3. | In line 214, about statistical significance at the 5% level, while I understand what you mean, it should be clear that the confidence level is 95% and that the decision to accept the null hypothesis or alternate hypothesis is based on the alpha value of 0.05. | The statistical analysis section has been revised to explicitly state that all hypothesis tests will be two-sided and conducted using a significance level (α) of 0.05, corresponding to a 95% confidence level. We have also revised the wording to avoid the common misconception of "accepting" the null hypothesis. | Methodology  Page 10 – 11 |
| 4. | Similar to Aim 1, the reviewers might find it difficult to include age-appropriate studies in the scoping review. WHO currently uses 19 years as a threshold and has ranges that you could find more informative to describe the terms "neonate," "child," and "adolescent" more clearly for the reviewers. | To improve consistency and transparency, we have included in the introduction section definition of children for this study inline with WHO and United Nations guidance. | Introduction  Page 5 |
| 5. | Aim 2 was clear, but I believe you could emphasize that the panel of experts will validate the findings from Aim 2. Recent scoping review protocols usually add a validation step, which I think is useful. The Delphi approach also achieves a similar validation intent. | We agree that explicitly linking the scoping review findings to the subsequent Delphi process strengthens the methodological coherence of the study.  The manuscript has therefore been revised to clarify that the findings from the scoping review will serve as the evidence base presented to the expert panel during the modified Delphi process. | Methodology  Page 17 |
