# Supplementary material for: Investigating strategies to enhance access to HIV services among children in Malawi: A multifaceted research approach, study protocol
Source: PLoS One. 2026 Sep 3;21(9):e0357408. doi: 10.1371/journal.pone.0357408 (PMC13541177; doi:10.1371/journal.pone.0357408)
Supplement: Peer review history

# Author comment: Author response to Decision Letter 0

**DOI:** 10.1371/journal.pone.0357408.r001

**Transfer Alert**

This paper was transferred from another journal. As a result, its full editorial history (including decision letters, peer reviews and author responses) may not be present.

26 Mar 2026

# Peer review: Decision Letter 0

**DOI:** 10.1371/journal.pone.0357408.r002

**Authors:** Amos Buh

29 Jun 2026

Dear Dr. Matola,

Thank you for submitting your manuscript to PLOS ONE. After careful consideration, we feel that it has merit but does not fully meet PLOS ONE’s publication criteria as it currently stands. Therefore, we invite you to submit a revised version of the manuscript that addresses the points raised during the review process.

Please submit your revised manuscript by Aug 13 2026 11:59PM. If you will need more time than this to complete your revisions, please reply to this message or contact the journal office at plosone@plos.org. When you're ready to submit your revision, log on to https://www.editorialmanager.com/pone/ and select the 'Submissions Needing Revision' folder to locate your manuscript file.

- A letter that responds to each point raised by the academic editor and reviewer(s). You should upload this letter as a separate file labeled 'Response to Reviewers'.
- A marked-up copy of your manuscript that highlights changes made to the original version. You should upload this as a separate file labeled 'Revised Manuscript with Track Changes'.
- An unmarked version of your revised paper without tracked changes. You should upload this as a separate file labeled 'Manuscript'.

If you would like to make changes to your financial disclosure, please include your updated statement in your cover letter. Guidelines for resubmitting your figure files are available below the reviewer comments at the end of this letter.

If applicable, we recommend that you deposit your laboratory protocols in protocols.io to enhance the reproducibility of your results. Protocols.io assigns your protocol its own identifier (DOI) so that it can be cited independently in the future. For instructions see: https://journals.plos.org/plosone/s/submission-guidelines#loc-laboratory-protocols. Additionally, PLOS ONE offers an option for publishing peer-reviewed Lab Protocol articles, which describe protocols hosted on protocols.io. Read more information on sharing protocols at https://plos.org/protocols?utm_medium=editorial-email&utm_source=authorletters&utm_campaign=protocols.

As the corresponding author, your ORCID iD is verified in the submission system and will appear in the published article. PLOS supports the use of ORCID, and we encourage all coauthors to register for an ORCID iD and use it as well. Please encourage your coauthors to verify their ORCID iD within the submission system before final acceptance, as unverified ORCID iDs will not appear in the published article. *Only*  the individual author can complete the verification step; PLOS staff *cannot*  verify ORCID iDs on behalf of authors.

We look forward to receiving your revised manuscript.

Kind regards,

Amos Buh, BSc., MPH, PhD

Academic Editor

PLOS One

Journal Requirements:

When submitting your revision, we need you to address these additional requirements.

1. Please ensure that your manuscript meets PLOS ONE's style requirements, including those for file naming. The PLOS ONE style templates can be found at

https://journals.plos.org/plosone/s/file?id=wjVg/PLOSOne_formatting_sample_main_body.pdf and

https://journals.plos.org/plosone/s/file?id=ba62/PLOSOne_formatting_sample_title_authors_affiliations.pdf

2. Thank you for stating the following in your Competing Interests section: [No competing interests].

Please complete your Competing Interests on the online submission form to state any Competing Interests. If you have no competing interests, please state ""The authors have declared that no competing interests exist."", as detailed online in our guide for authors at http://journals.plos.org/plosone/s/submit-now

This information should be included in your cover letter; we will change the online submission form on your behalf.

3. Please provide a complete Data Availability Statement in the submission form, ensuring you include all necessary access information or a reason for why you are unable to make your data freely accessible. If your research concerns only data provided within your submission, please write "All data are in the manuscript and/or supporting information files" as your Data Availability Statement.

4. Your ethics statement should only appear in the Methods section of your manuscript. If your ethics statement is written in any section besides the Methods, please delete it from any other section.

5. If the reviewer comments include a recommendation to cite specific previously published works, please review and evaluate these publications to determine whether they are relevant and should be cited. There is no requirement to cite these works unless the editor has indicated otherwise.

Additional Editor Comments:

Please address all reviewers comments.

[Note: HTML markup is below. Please do not edit.]

Reviewers' comments:

Reviewer's Responses to Questions

**Comments to the Author**

1. Does the manuscript provide a valid rationale for the proposed study, with clearly identified and justified research questions?

Reviewer #1: Partly

Reviewer #2: Yes

Reviewer #3: Yes

**********

2. Is the protocol technically sound and planned in a manner that will lead to a meaningful outcome and allow testing the stated hypotheses?

Reviewer #1: Partly

Reviewer #2: Yes

Reviewer #3: Yes

**********

3. Is the methodology feasible and described in sufficient detail to allow the work to be replicable?

Reviewer #1: No

Reviewer #2: Yes

Reviewer #3: Yes

**********

4. Have the authors described where all data underlying the findings will be made available when the study is complete?

The PLOS Data policy

Reviewer #1: No

Reviewer #2: Yes

Reviewer #3: Yes

**********

5. Is the manuscript presented in an intelligible fashion and written in standard English?

Reviewer #1: No

Reviewer #2: Yes

Reviewer #3: Yes

**********

Please use the space provided to explain your answers to the questions above and, if applicable, provide comments about issues authors must address before this protocol can be accepted for publication. You may also include additional comments for the author, including concerns about research or publication ethics.

You may also provide optional suggestions and comments to authors that they might find helpful in planning their study.

Reviewer #1: Overall assessment

This manuscript addresses an important and policy-relevant public health problem: persistent inequities in access to HIV services among children in Malawi despite substantial national progress in adult HIV epidemic control. The topic is timely, relevant, and potentially suitable for PLOS ONE as a protocol manuscript.

The proposed multi-phase approach combining secondary epidemiological analysis, scoping review, expert consensus development, and feasibility assessment has conceptual merit. However, in its current form, the manuscript has major methodological, structural, and reporting weaknesses that substantially limit confidence in its scientific rigor, reproducibility, and suitability for publication.

The most significant concerns relate to protocol coherence, internal inconsistencies regarding study status, insufficient methodological precision, weak justification for the integrated design, concerns regarding the feasibility assessment methodology, and incomplete adherence to PLOS reporting expectations.

Major comments

1. Study rationale and research question

The manuscript addresses a valid and important academic and public health problem. The disparity between adult and paediatric HIV outcomes in Malawi provides a strong rationale for the study .

However, the research question remains too broad and insufficiently operationalized. Terms such as “access” and “approaches” are not clearly defined, making the precise scope of inquiry unclear. It is not explicit whether the study focuses primarily on HIV testing, diagnosis, ART initiation, retention in care, viral suppression, health systems strengthening, or implementation strategies.

The manuscript would also benefit from a stronger justification for the claimed evidence gap, particularly the assertion that there is limited systematic evidence on effective interventions in this area.

In addition, the rationale for combining four distinct methodological studies into a single protocol is not sufficiently articulated. While the individual components are understandable, the manuscript does not clearly explain the overarching logic linking:

* national epidemiological analysis,

* evidence synthesis,

* Delphi consensus development,

* and feasibility assessment.

A clear conceptual workflow or logic model is needed.

2. Protocol coherence and study status

A major concern is inconsistency regarding the protocol’s status.

This manuscript is presented as a prospective study protocol, yet:

* Sub-study 1 is described as already completed and published.

* Sub-study 2 appears partially completed.

* Other components remain prospective.

This creates substantial ambiguity regarding whether this is:

* a true protocol,

* a hybrid progress report,

* or a doctoral programme methods overview.

Completed work should not be presented as future planned research without clear explanation.

3. Technical soundness and methodological rigor

The protocol includes substantial methodological description, but important weaknesses reduce confidence in its technical robustness.

Key concerns:

* insufficient detail regarding analytic decision-making;

* unclear outcome definitions;

* limited explanation of data integration procedures;

* absence of clear distinction between confirmatory and exploratory analyses;

* inadequate justification for some design choices.

The protocol appears conceptually ambitious but methodologically uneven.

Section-specific comments

4. Sub-study 1: Secondary quantitative analysis

This component raises several concerns.

First, because this study is already described as completed and published, it should not be framed as prospective protocol work.

Second, the statistical analysis plan lacks sufficient rigor. The manuscript proposes descriptive analyses and proportion comparisons , but the dataset spans more than a decade, suggesting the need for stronger trend analysis methods.

Third, key methodological details are missing:

* precise variable definitions;

* unit of analysis;

* treatment of missing data;

* reconciliation between DHAMIS programme data and Spectrum estimates;

* assumptions underlying denominator construction.

Without these details, reproducibility is limited.

5. Sub-study 2: Scoping review

The scoping review methodology is broadly structured but contains methodological inconsistencies.

Concerns include:

* mixing multiple frameworks (Arksey & O’Malley, Levac, JBI, PRISMA-P, PRISMA-ScR) without clear methodological coherence;

* inappropriate inclusion of formal risk-of-bias tools, which is not standard for scoping reviews unless explicitly justified;

* inconsistency between PCC and PICO frameworks;

* mismatch between stated target population (children 0–14 years) and inclusion of adolescents in search terms;

* unsupported dismissal of language bias resulting from English-only inclusion.

This component requires methodological tightening.

6. Sub-study 3: Modified e-Delphi process

The Delphi methodology requires greater precision.

Specific concerns:

* unclear expert eligibility criteria;

* reliance on professional networks, creating potential selection bias;

* ambiguous consensus definitions;

* no clearly defined stopping rule for Delphi rounds;

* insufficient detail on questionnaire development and piloting;

* risk that the planned in-person workshop compromises Delphi anonymity and independence.

These methodological details should be clarified to improve rigor and reproducibility.

7. Sub-study 4: Feasibility and acceptability assessment

This is the weakest methodological component.

The proposed design assesses participant perceptions after watching a short video illustrating interventions rather than assessing interventions under real implementation conditions .

This raises major validity concerns.

Watching a simulated intervention does not constitute genuine feasibility testing. Participants cannot reliably assess:

* operational feasibility,

* implementation burden,

* workflow integration,

* sustainability,

* staffing implications.

At best, this design assesses perceived acceptability, appropriateness, or hypothetical feasibility.

Additional concerns:

* unclear mixed-methods integration;

* weak sample size justification;

* single-site design without adequate justification;

* high risk of social desirability bias;

* principal investigator serving as sole data collector, increasing bias risk.

This component requires substantial redesign or clearer reframing.

8. Replicability and methodological transparency

The manuscript provides moderate methodological detail, but reproducibility remains limited.

Missing or insufficiently described elements include:

* sample size calculations or power analysis for quantitative work;

* justification for participant numbers in Delphi and feasibility studies;

* instrument validation procedures;

* data handling procedures;

* bias mitigation strategies;

* integration methods for mixed-methods findings.

More methodological precision is needed for another researcher to replicate the work.

9. Data availability

The manuscript does not adequately meet PLOS ONE data-sharing expectations.

Although this is a protocol manuscript, the authors should clearly specify how data generated from the study will be shared upon completion.

Current concerns:

* no named public repository;

* no description of what datasets will be shared;

* no clarification of access restrictions for national programme data;

* no plan for sharing de-identified qualitative data.

A PLOS-compliant data availability statement is required.

10. Ethics

Ethics reporting is inconsistent.

The manuscript states ethical approval “will be obtained,” while submission metadata suggests approval already exists.

Additional concerns:

* ambiguity regarding secondary data ethics approval versus exemption;

* confidentiality risks associated with recruitment through HIV clinic settings;

* no explicit governance for use of participant actors in intervention videos.

These issues should be clarified.

11. Language, presentation, and clarity

The manuscript is generally understandable, but substantial editing is required before publication consideration.

Concerns:

* grammatical and typographical errors;

* awkward sentence construction;

* excessive repetition;

* inconsistent terminology;

* formatting inconsistencies;

The manuscript would benefit from thorough language editing.

Minor comments

* Define DHAMIS at first use.

* Clarify whether adolescents are included.

* Justify selection of Kabudula Community Hospital.

* Clarify whether adapted AIM/IAM/FIM tools retain validation properties.

* Standardize terminology throughout.

* Improve formatting consistency.

* Remove speculative findings language from protocol sections.

* Strengthen limitations discussion.

* Provide clearer summary workflow figure.

Reviewer #2: the protocal is sound, has clear rationale, multi method design combining secondary data review and literature review, is aligned with global target, has mentioned clear approval, consent and confidentality demonstrated. Its based on Malawi with a possibility of it being scaled. However theres need to condence the abtract, could also mention the exixting strategies which have failed if any. For methods study 1but justification for choice of tests (chi-square vs Fisher’s exact) could be clearer.Sub-study 2: Scoping review lacks detail on inclusion/exclusion criteria, databases, and search strategy.Sub-study 3: The modified e-Delphi process is appropriate, but criteria for expert selection should be specified.Sub-study 4: Feasibility assessment mentions video demonstration and questionnaires, but lacks detail on sample size, recruitment, and analytic approach

data availability written as N/A since its a protocal but may give a statement on how data will be shared

Reviewer #3: Dear Author,

The research question is relevant, and you clearly described the current disparities and how the pediatric population is impacted. I have a few comments.

1. For Aim 1, in line 197, what informed the decision to disaggregate data into 15 and below and above 15 years old? Does adult age begin at 15? I'd recommend using WHO standardized ages and age boundaries that are less wide for a more granular comparison.

2. In line 203, why is the study team using both SPSS and Stata? I'd recommend sticking with one tool to ease collaboration among the analysts within the team.

3. In line 214, about statistical significance at the 5% level, while I understand what you mean, it should be clear that the confidence level is 95% and that the decision to accept the null hypothesis or alternate hypothesis is based on the alpha value of 0.05.

4. Similar to aim 1, the reviewers might find it difficult to include age-appropriate studies in the scoping review. WHO currently uses 19 years as a threshold and has ranges that you could find more informative to describe the terms "neonate," "child," and "adolescent" more clearly for the reviewers.

5. Aim 2 was clear, but I believe you could emphasize that the panel of experts will validate the findings from Aim 2. Recent scoping review protocols usually add a validation step, which I think is useful. The Delphi approach also achieves a similar validation intent.

Looking forward to reading the revised edition.

**********

what does this mean?). If published, this will include your full peer review and any attached files.

If you choose “no”, your identity will remain anonymous but your review may still be made public.

**Do you want your identity to be public for this peer review?**  For information about this choice, including consent withdrawal, please see our Privacy Policy

Reviewer #1: No

Reviewer #2: No

Reviewer #3: **Yes:**  Adebowale Sylvester Adeyemi

**********

[NOTE: If reviewer comments were submitted as an attachment file, they will be attached to this email and accessible via the submission site. Please log into your account, locate the manuscript record, and check for the action link "View Attachments". If this link does not appear, there are no attachment files.]

To ensure your figures meet our technical requirements, please review our figure guidelines: https://journals.plos.org/plosone/s/figures

You may also use PLOS’s free figure tool, NAAS, to help you prepare publication quality figures: https://journals.plos.org/plosone/s/figures#loc-tools-for-figure-preparation.

NAAS will assess whether your figures meet our technical requirements by comparing each figure against our figure specifications.

# Author comment: Author response to Decision Letter 1

**DOI:** 10.1371/journal.pone.0357408.r003

16 Jul 2026

Dear Editor,

On behalf of my co-authors, I am pleased to submit the revised version of our manuscript entitled " Investigating strategies to enhance access to HIV services among children in Malawi: A multifaceted research approach, study protocol " for further consideration for publication in PLOS ONE.

We thank you and the reviewers for the thorough and constructive evaluation of our manuscript. We greatly appreciate the insightful comments, which have substantially strengthened the scientific rigor, methodological transparency, and overall clarity of the protocol. We have carefully considered every comment raised by the reviewers and have revised the manuscript accordingly. A detailed point-by-point response to all reviewer comments accompanies this submission.

The major revisions undertaken include, but are not limited to, the following:

• The study rationale and research question have been refined by clearly defining the concepts of "access to HIV services" and "approaches," strengthening the justification for the evidence gap, and articulating the conceptual linkage between the four sub-studies through a sequential mixed-methods framework supported by a workflow figure.

• The manuscript has been revised to clearly distinguish completed work from prospective study components, thereby resolving ambiguities regarding the protocol status.

• Methodological rigor has been strengthened across all study components through expanded descriptions of study variables, outcome definitions, statistical analyses, data integration procedures, sampling strategies, instrument development, consensus criteria, and bias mitigation measures.

• The scoping review methodology has been aligned with current Joanna Briggs Institute guidance and the PRISMA Extension for Scoping Reviews (PRISMA-ScR), with consistent application of the Population–Concept–Context framework and clarification of eligibility criteria and search strategy.

• The modified Delphi methodology has been expanded to include explicit expert eligibility criteria, consensus definitions, stopping rules, questionnaire development procedures, and clarification of the role of the post-Delphi stakeholder workshop.

• The feasibility component has been reframed to evaluate anticipated feasibility, acceptability, and appropriateness rather than real-world implementation feasibility, with additional detail provided regarding sampling, recruitment, mixed-methods analysis, and study limitations.

• Additional information has been incorporated regarding data management, ethical considerations, methodological transparency, and study limitations to improve reproducibility and compliance with reporting standards.

• The manuscript has undergone comprehensive language editing to improve clarity, consistency, grammar, terminology, and formatting throughout.

In response to the journal's editorial requirements, we also wish to clarify the following:

1. PLOS ONE formatting requirements

We confirm that the manuscript has been revised to comply with the PLOS ONE formatting guidelines, including formatting of the title page, manuscript structure, headings, tables, figures, references, and file naming conventions in accordance with the journal's style templates.

2. Competing Interests

The authors declare that no competing interests exist.

3. Data Availability Statement

As this manuscript describes a study protocol, no datasets have yet been generated or analysed.

The revised manuscript now includes the following Data Availability Statement:

"As this manuscript describes a study protocol, no datasets have yet been generated or analysed. Upon completion of the study, de-identified quantitative datasets supporting the published findings will be deposited in an appropriate public data repository where permitted by national data-sharing agreements. Qualitative data will be shared in a de-identified form upon reasonable request, subject to ethical approval, participant confidentiality, and Malawi Ministry of Health data governance requirements. Secondary national programme data used in this study remain subject to Ministry of Health data access policies and cannot be shared by the authors without permission from the data custodian."

4. Ethics Statement

We confirm that the ethics statement now appears only within the Methods section of the manuscript, in accordance with PLOS ONE requirements, and has been removed from all other sections.

5. Reviewer-recommended references

We carefully evaluated all publications suggested by the reviewers. Relevant methodological and substantive references have been incorporated where appropriate to strengthen the scientific justification and reporting of the study. References that were not directly relevant to the study objectives or methodology were not included.

We believe that the revisions have substantially strengthened the manuscript and have addressed all concerns raised by both the reviewers and the editorial office. We hope that the revised manuscript is now suitable for publication in PLOS ONE.

Thank you for your time and consideration. We look forward to your favourable response.

Attachment Submitted filename: Response_to_reviewers.docx

# Peer review: Decision Letter 1

**DOI:** 10.1371/journal.pone.0357408.r004

**Authors:** Amos Buh

17 Aug 2026

Investigating strategies to enhance access to HIV services among children in Malawi: a multifaceted research approach, study protocol

PONE-D-26-13065R1

Dear Dr. Matola,

We’re pleased to inform you that your manuscript has been judged scientifically suitable for publication and will be formally accepted for publication once it meets all outstanding technical requirements.

Within one week, you’ll receive an e-mail detailing the required amendments. When these have been addressed, you’ll receive a formal acceptance letter and your manuscript will be scheduled for publication.

An invoice will be generated when your article is formally accepted. Please note, if your institution has a publishing partnership with PLOS and your article meets the relevant criteria, all or part of your publication costs will be covered. Please make sure your user information is up-to-date by logging into Editorial Manager at Editorial Manager® and clicking the ‘Update My Information' link at the top of the page. For questions related to billing, please contact billing support.

If your institution or institutions have a press office, please notify them about your upcoming paper to help maximize its impact. If they’ll be preparing press materials, please inform our press team as soon as possible -- no later than 48 hours after receiving the formal acceptance. Your manuscript will remain under strict press embargo until 2 pm Eastern Time on the date of publication. For more information, please contact onepress@plos.org.

Kind regards,

Amos Buh, BSc., MPH, PhD

Academic Editor

PLOS One

Additional Editor Comments (optional):

Reviewers' comments:

Reviewer's Responses to Questions

**Comments to the Author**

1. Does the manuscript provide a valid rationale for the proposed study, with clearly identified and justified research questions?

Reviewer #2: Yes

Reviewer #3: Yes

**********

2. Is the protocol technically sound and planned in a manner that will lead to a meaningful outcome and allow testing the stated hypotheses?

Reviewer #2: Yes

Reviewer #3: Yes

**********

3. Is the methodology feasible and described in sufficient detail to allow the work to be replicable?

Reviewer #2: Yes

Reviewer #3: Yes

**********

4. Have the authors described where all data underlying the findings will be made available when the study is complete?

The PLOS Data policy

Reviewer #2: Yes

Reviewer #3: Yes

**********

5. Is the manuscript presented in an intelligible fashion and written in standard English?

Reviewer #2: Yes

Reviewer #3: Yes

**********

Please use the space provided to explain your answers to the questions above and, if applicable, provide comments about issues authors must address before this protocol can be accepted for publication. You may also include additional comments for the author, including concerns about research or publication ethics.

You may also provide optional suggestions and comments to authors that they might find helpful in planning their study.

Reviewer #2: As a study protocol, the multi-methodological approach is clearly aligned with the core research question. The descriptive and comparative statistics planned are appropriate and well-defined and Ethical approval sources are detailed, and data governance policies are appropriately stated.

Reviewer #3: It was great reading through the document once again. The revised copy adequately addressed my previous concerns.

**********

what does this mean?). If published, this will include your full peer review and any attached files.

If you choose “no”, your identity will remain anonymous but your review may still be made public.

**Do you want your identity to be public for this peer review?**  For information about this choice, including consent withdrawal, please see our Privacy Policy

Reviewer #2: No

Reviewer #3: **Yes:**  Adebowale Sylvester Adeyemi

**********

# Editor report: Acceptance letter

**DOI:** 10.1371/journal.pone.0357408.r005

**Authors:** Amos Buh

PONE-D-26-13065R1

PLOS One

Dear Dr. Matola,

I'm pleased to inform you that your manuscript has been deemed suitable for publication in PLOS One. Congratulations! Your manuscript is now being handed over to our production team.

At this stage, our production department will prepare your paper for publication. This includes ensuring the following:

* All references, tables, and figures are properly cited

* All relevant supporting information is included in the manuscript submission,

* There are no issues that prevent the paper from being properly typeset

You will receive further instructions from the production team, including instructions on how to review your proof when it is ready. Please keep in mind that we are working through a large volume of accepted articles, so please give us a few days to review your paper and let you know the next and final steps.

Lastly, if your institution or institutions have a press office, please let them know about your upcoming paper now to help maximize its impact. If they'll be preparing press materials, please inform our press team within the next 48 hours. Your manuscript will remain under strict press embargo until 2 pm Eastern Time on the date of publication. For more information, please contact onepress@plos.org.

You will receive an invoice from PLOS for your publication fee after your manuscript has reached the completed accept phase. If you receive an email requesting payment before acceptance or for any other service, this may be a phishing scheme. Learn how to identify phishing emails and protect your accounts at https://explore.plos.org/phishing.

If we can help with anything else, please email us at customercare@plos.org.

Thank you for submitting your work to PLOS One and supporting open access.

Kind regards,

PLOS One Editorial Office Staff

on behalf of

Dr. Amos Buh

Academic Editor

PLOS One
